# Avoiding the Misuse of Pathway Analysis Tools in Environmental Metabolomics

**DOI:** 10.1021/acs.est.2c05588

**Published:** 2022-09-26

**Authors:** Cecilia Wieder, Jacob G. Bundy, Clément Frainay, Nathalie Poupin, Pablo Rodríguez-Mier, Florence Vinson, Juliette Cooke, Rachel P. J. Lai, Fabien Jourdan, Timothy M. D. Ebbels

**Affiliations:** † Department of Metabolism, Digestion and Reproduction, Faculty of Medicine, Imperial College London, Burlington Danes Building, Du Cane Road, London W12 0NN, U.K.; ‡ Toxalim (Research Centre in Food Toxicology), Université de Toulouse, INRAE, ENVT, INP-Purpan, UPS, 180 chemin de Tournefeuille St-Martin-du-Touch, BP 3, 31931 Toulouse Cedex, France; § Department of Infectious Disease, Faculty of Medicine, Commonwealth Building, Imperial College London, Du Cane Road, London W12 0NN, U.K.; ∥ MetaToul-MetaboHUB, National Infrastructure of Metabolomics and Fluxomics, 180 chemin de Tournefeuille St-Martin-du-Touch, BP 3, 31931 Toulouse Cedex, France

**Keywords:** metabolomics, metabonomics, environmental metabolomics, metabolic pathway, enrichment analysis, pathway analysis, over-representation analysis

Within the past 20 years, metabolomics has moved from an exciting innovation within the environmental sciences to something that is almost routine. It can be considered as a means to generate metabolite biomarkers, although it is also important to note the cogent criticisms of the environmental biomarker approach that have been made within ecotoxicology: briefly, that biomarkers are surrogates for macro phenotypes (e.g., survival, reproduction, and behavior) that have population-level effects and that it is generally more straightforward and meaningful to measure these end points directly.^1^ Some studies have emphasized instead the ability to gain potentially relevant mechanistic information, even for nonmodel organisms, especially when used as part of a multiomic approach.^2^ An improved biological understanding is often implicitly or explicitly part of the justification of including metabolomics in a study.

So far, so good, but there is a problem: there is no simple, universally accepted way of reverse engineering mechanistic understanding from metabolomic data, even for model organisms, and the problem is even more complicated for nonmodel species. The closest thing to a standard approach is pathway analysis (PA), i.e., making use of existing biochemical knowledge. There are multiple approaches to PA, but we will focus on just one, over-representation analysis (ORA). (NB that the term ORA is often not used, and many authors refer generically to “pathway enrichment” methods.) It should clearly be understood, though, that ORA is certainly not the only approach to analyzing metabolomics data. It is beyond the scope of this work to review the options available, but we direct the interested reader to recent reviews.^3,4^ ORA uses the intuitive approach of identifying metabolite biomarker “hits” and comparing them to the numbers of metabolites in specific pathways, to determine if there are either more or fewer hits than one would expect by chance. It therefore has the twin advantages of being simple to calculate and simple to understand. It does, though, have disadvantages. One potential limitation is shared with all methods that rely on predetermined pathway definitions: traditional pathways are, generally, subjective and heuristic approaches to imposing order on a biochemical network.^5^ While this is an important point, we will simply note it here and pass on, and bear in mind that “pathways” are, at least to some extent, arbitrary definitions. The problem is exacerbated for nonmodel organisms, in that accurate metabolic pathway definitions may not be available. It should also be noted that metabolites may contribute to many different pathways: for example, glucose is present in 23 of 263 pathways (KEGG, human), and ATP is present in 880 of 1669 pathways (Reactome, human). Just because a metabolite may be part of a particular pathway, then, does not mean that changes in that metabolite necessarily mean changes in that pathway. Particular care must be taken with environmental organisms not to misinterpret changes with respect to examples from human medicine. A second obvious limitation of ORA is that the criteria for defining significant metabolites are also arbitrary, usually, but not necessarily, based on selecting a threshold for *P* values from null hypothesis significance testing.

It is also possible to draw incorrect conclusions from ORA. For instance, the online Metaboanalyst web server provides a suite of tools for metabolomic analysis, including, but not limited to, ORA.^6^ These have become justly popular, as they are free to use, available online, integrated with data processing and biostatistical modules, and updated to ensure they remain current. They also provide some opportunities to set parameters that affect the results, opening up the possibility of inadvertently misusing the tools. (NB that this is not an implicit criticism of the team behind Metaboanalyst: individual researchers should take responsibility for their own results, including interpretation.) We recently published a study of the sensitivity of ORA of metabolomics data to some of the different parameters that can be chosen.^7^ A wide range of different factors affect the results (Figure 1). First, and obviously, the choice of database and pathway definitions has a major impact. Second, the precise *P* value cutoff used for selecting metabolite hits had, unsurprisingly, major effects on the number of significantly enriched pathways. Third, using a background or reference metabolome (i.e., the total list of annotated metabolites detected in a particular experiment) is critically important: if the background is not taken into consideration, the results tend to be very overoptimistic—the *P* values obtained are much more significant than they should be.

**Figure 1:**
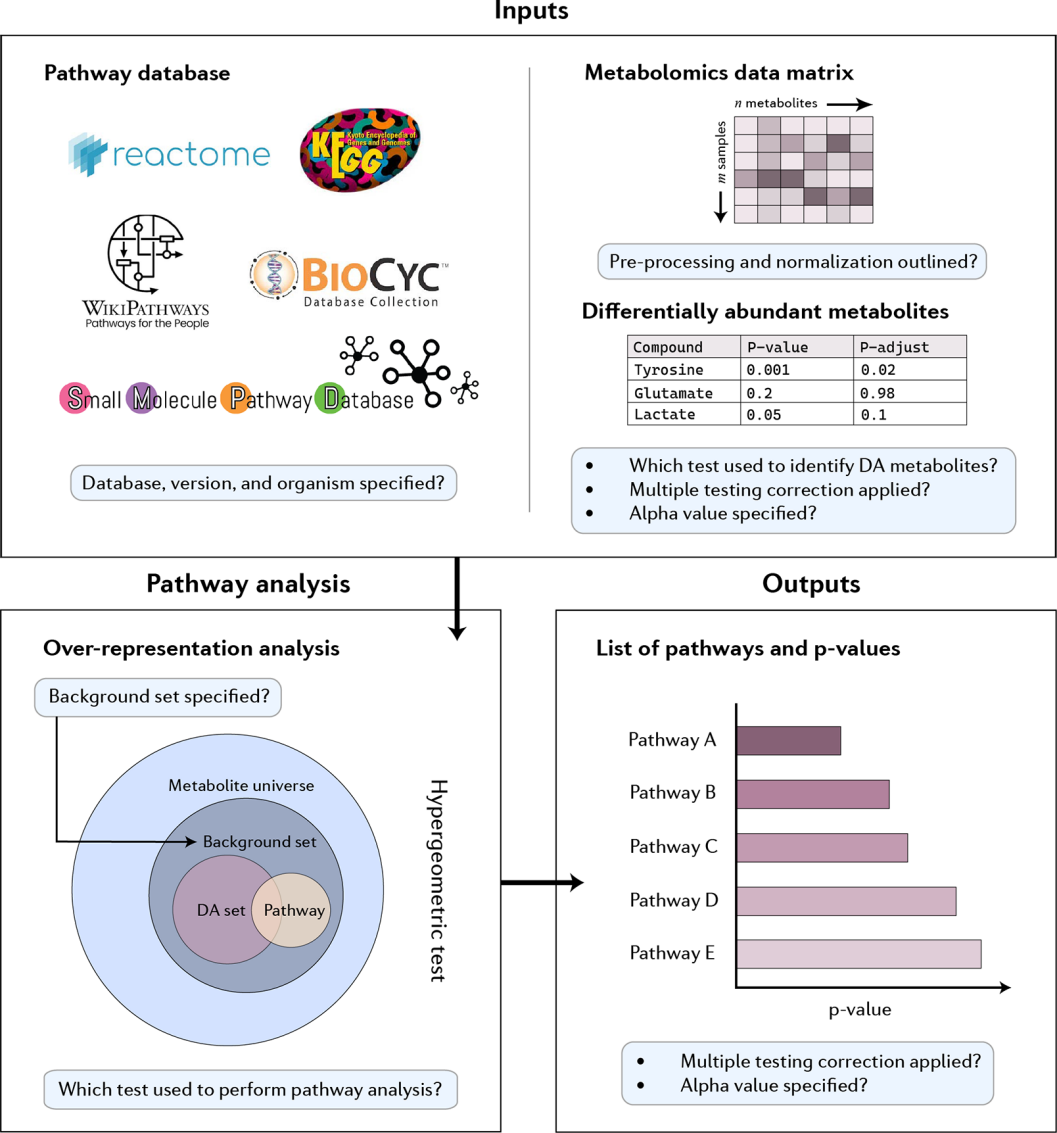
Schematic illustration of the factors that can affect metabolic pathway analysis of metabolomics data, at different stages of the study. Inputs: affected by the organism and pathway database chosen; affected by the significance threshold used to choose the number of metabolite hits. Pathway analysis: the use of a background set (reference metabolome) is particularly important. Outputs: have the *P* values for the pathways been corrected for multiple testing (based on the total number of pathways in the database)?

We decided to survey the literature to get an idea of the current practice in environmental metabolomics. We searched for environmental metabolomics papers (Clarivate Web of Science core database, July 7, 2022; searched all fields for “metabolom* or metabonom*”, and constrained by Topic = Environmental Sciences, by Document Type = Article, and by Publication Year = 2020–2022) and identified 988 recent papers. We randomly selected 30 papers from this list (after manually excluding three more reviews that had been incorrectly labeled; the list of papers is given in Table S1) and checked to see what form of PA, if any, was used. Two-thirds of the studies (20 of 30) employed PA (two additional studies mapped metabolites to pathways, but without an associated statistical test); all of these (20 of 20) used ORA, although this generally was not specified by name. Two of them used simultaneous enrichment of transcriptomic and metabolomic data, although full details were not given. Fourteen of the studies used Metaboanalyst; one used the R package Mummichog, and seven failed to specify which software was used. With the exception of the study that had used Mummichog, they generally failed in reporting key parameters that affect the outcome. No studies specified exactly which pathway database was used for the analysis, including for which organism; eight mentioned KEGG pathways but with no more detail given. No studies reported if they corrected for multiple testing in the software output (i.e., based on the number of different metabolic pathways tested); several used plots including an uncorrected *P* value scale with no additional information given. No studies made any mention of a reference or background metabolome set. Some studies set ad hoc thresholds based on the “pathway impact” statistic provided by Metaboanalyst. It is clear that ORA is being unintentionally misused in environmental metabolomics research, in a fashion that is likely to lead to misleading results.

We conclude by making some brief recommendations for using ORA with environmental metabolomics data (see ref (7) for more detail and fuller discussion).

(1) Accurately report the analyses carried out. The specific software package/online tool used should be reported, along with all of the parameters, even if they were left as defaults, including the database version and organism used for pathways. Specify what *P* value cutoff/other parameter was used for selection of metabolite hits for PA, including any correction for multiple testing, and also whether correction for multiple testing was carried out on the output (i.e., based on the number of pathways).

(2) Always upload a reference metabolome, or “background set”. In other words, the list of all metabolites that have been identified in that specific study. If this is not done, the results should be treated with extreme caution, as they may inaccurately identify pathways as significantly enriched.

(3) Avoid definitive statements about which pathways have been impacted in a particular study. This type of pathway-based approach is, ideally, used to help generate hypotheses that can then be validated by independent experiments; even if further experiments are not feasible, the limitations should be appreciated.

We hope these simple recommendations should help researchers avoid some of the common errors that currently plague environmental metabolomics research.

## Supporting Information Available

A list of environmental metabolomics papers used to investigate real-world pathway analysis approaches in environmental metabolomics (Table S1) (XLSX)
